# Social and behavioural risk factors in the prevention and management of cardiovascular disease in Kerala, India: a catchment area population survey

**DOI:** 10.1186/s12872-020-01595-x

**Published:** 2020-07-08

**Authors:** Saju Madavanakadu Devassy, Martin Webber, Lorane Scaria, Jotheeswaran Amuthavalli Thiyagarajan, Meredith Fendt-Newlin, Jacques Joubert, Anuja Maria Benny, Anjana Nannatt, Lynette Joubert

**Affiliations:** 1 Rajagiri College of Social Sciences (Autonomous), Rajagiri P. O, Kalamassery, Kochi, Kerala 683 104 India; 2 International Centre for Mental Health Social Research, Department of Social Policy and Social Work, University of York, York, Heslington YO10 5DD UK; 3 Department of Ageing and Life Course, World Health Organization, Geneva, Switzerland; 4 Institute of Psychiatry, Psychology and Neuroscience, King’s College London, London, UK; 5 World Health Organization, Ankara, Turkey; 6 University of Melbourne, Melbourne, Australia

**Keywords:** Hypertension, Diabetes, Common mental health problems, India, Social risk

## Abstract

**Background:**

Cardiovascular disease (CVD) is the leading cause of mortality in India. Social and behavioural factors are strongly interrelated in the prevention and control of CVD. The ability to make lifestyle changes to control hypertension and diabetes (major risk factors for CVD) is determined by factors such as education, gender, caste, poverty, and urbanicity. This study aimed to improve our understanding of the inter-relationship of social and behavioural factors in the management of elevated serum glucose and high blood pressure and co-morbid mental health conditions.

**Methods:**

A population-based catchment area cross sectional survey was conducted in Kerala, India. Data were collected from residents aged over 30 years (*n* = 997) using standardized tools and clinical measures. We performed latent class analysis incrementally to extract homogeneous latent classes of individuals based on their responses to social and behavioural risk factors in the survey. Using structural equation models, we assessed the mediating effect of depression and anxiety, and social or behavioural risk factors, on management of high blood pressure and raised serum glucose levels.

**Results:**

The prevalence of high blood pressure and blood glucose in the sample was 33 and 26% respectively. Latent class analysis found three clusters of risk factors. One had a predominance of behavioural characteristics, another of social risk factors and the third was a low risk group. Age, female sex, and marital status had an effect on high blood pressure and high glucose, though were mediated by mental health, social and behavioural risk factors.

**Conclusions:**

Interventions to improve the management of risk factors for CVD need to address social risk factors and be sensitive to the needs of population sub-groups that may require additional support to access health services. An integration of social and health services may be required to achieve this.

## Background

Non-communicable diseases (NCDs) result in two thirds of deaths worldwide, are a major cause of chronic disability [1], and are a major health and economic concern. In India, cardiovascular disease (CVD) is currently the leading cause of mortality and is responsible for 28% of all deaths [2]. Every fourth and ninth individual in India aged above 18 years has high blood pressure and elevated blood glucose, respectively [3]. The most important behavioural risk factors related to CVD prevalent in India are lack of physical activity, tobacco use, and excess use of alcohol [4]. These negative behaviours are frequently present in people with hypertension, glucose intolerance and obesity. Moreover, socioeconomic status, both affluence and poverty, have recognized association with NCDs.

There is a recognized strong association between social and behavioural factors in both the prevalence and management of NCDs, particularly CVD. For example, a higher prevalence of raised blood pressure is found among young urban men and poorer households [5–7]. The rapidly improving socioeconomic status in India is associated with an increased NCD risk. This may be due to a reduction of physical activity and increased rates of obesity and diabetes [4, 8, 9]. Diabetes Mellitus increases along a positive social gradient, with a higher prevalence in the more affluent and better educated sections of the Indian population [10]. Poverty is often associated with underdiagnosis and underreporting of Diabetes Mellitus and this presents an ongoing challenge in both the detection and management of the condition [11]. Moreover, the gender disparity and income inequality are significant factors related to both diabetes and depression in Indian women [12].

Health outcomes in India have improved overall in the last decades but health continues to be strongly affected by factors such as education, gender, caste and wealth. Barriers to effective management of NCDs are out-of-pocket expenditures and geography and, because of these, the management of risk factors for NCDs is often suboptimal in India. One third of the urban Indian population is hypertensive, but in only 38% of these is the hypertension controlled [13]. In rural India the situation is worse with control only being achieved in 11% [13]. The epidemiological transition found in India, characterized by increasing urbanization and increased affluence, is related to the increase in degenerative and lifestyle-related diseases such as hypertension and diabetes [14]. Also, factors such as poverty, poor social networks and adverse life events all act as barriers to the effective management of NCDs such as hypertension and diabetes, as well as the commonly associated co-morbid mental health issues such as depression and anxiety [15].

A positive social environment and good social support have been shown to have a significant impact on compliance with the medical management of chronic disease [16]. Good social networks have also been found to be a significant protective factor for depression in a rural south Indian community [17]. A better understanding of social factors relating to NCD management, will enhance the ability of primary health care services in India to effectively address what has been described as a looming epidemic of NCDs.

The traditional medical risk factors for NCDs are well-documented. These include behavioural risk factors such as alcohol misuse and cigarette smoking [18]. A recent scoping review examining social risk factors as related to NCDs in India found only 10 studies where social factors were documented or considered in the context of NCDs [15]. This review found that factors such as demographics, poverty, social networks, life events, health barriers and health risk behaviours were associated with poor management of diabetes and hypertension, as well as the co-morbidities of depression and anxiety in India [15]. From this it is clear that there is currently a lack of data about the inter-relationship between social, behavioural and traditional risk factors in India.

We aimed to explore in this cross-sectional study in a South-Indian community the relationship between social and behavioural risk factors and the management of NCDs, in particular their effect on poor management of elevated serum glucose and hypertension as well as their relationship to the co-morbid mental health conditions depression and anxiety. We aimed to:

- identify social risk factors relating to the effective management of elevated serum glucose and hypertension as well as cco-morbid mental health conditions such as depression and anxiety;
- empirically examine the relationship between these social risk factors and health outcomes;
- and explore the social and behavioral risk factors for the poor management of NCDs.

## Methods

### Study design

A population-based, cross sectional survey was conducted between May and July 2018 with a geographically defined semi-urban community. The survey aimed to determine the point prevalence of blood glucose, hypertension and the mental health comorbidities of depression and anxiety and the relationship between risk factors (social, behavioural, and psychosocial) and self-management of chronic disease. The survey was conducted as part of a UK-India Education and Research Initiative (UKIERI) funded project.

### Setting

The study was conducted in Keezhmadu panchayat in Ernakulam, Kerala, in south India, a semi-rural area having mixed culture and different economic ranges. The catchment area included people with all levels of education with Hindus, Muslims and Christians living together in almost equal proportions. A mix of economic activity was present, including agricultural labour and professional occupations.

### Sample size

Our assumption based on secondary data analysis performed with the 2003 World Health Survey dataset for India, was that nearly 30% of the population in south India is expected to experience one or more aforementioned chronic conditions. Precision calculations indicated that an overall sample of *n* = 1000 from the site would allow an estimation of the prevalence of 30% of one or more conditions (diabetes, hypertension, depression, and anxiety) with an absolute precision of +/− 5% (Prince et al., 2007). Data were collected with a total sample size of 997 individuals, 365 were males and 632 females.

### Sampling

The participants were recruited in two phases. Firstly, a mapping exercise involving local officials in identification of the wards in Keezhmadu panchayat and a door-to-door visits in defined geographical catchment areas allowed the researchers to enumerate the number of eligible participants aged ≥30 in each household. Secondly, all individuals who fulfilled the age criteria from each household were invited to participate until the desired sample size was reached. Informed consent was obtained from each participant prior to interview and examination.

### Measures

The primary outcomes were management of diabetes, hypertension, depression and anxiety. The point prevalence of diabetes was estimated based on self-reported diagnosis and measurement of blood glucose. Additionally, all participants were screened for Type 2 diabetes mellitus and pre-diabetes using capillary blood obtained by finger-prick using a digital device (Free Style Opium Neo H Meter Kit). Blood sugar was measured regardless of the time of the last meal. Trained interviewers used a lancet for finger prick to obtain fresh capillary whole blood and a glucometer to measure the random glucose level. The International Diabetes Federation and WHO criterion was used to classify participants into diabetic or normal. A blood glucose level between 79 and 200 mg/dl was considered normal and > 200 mg/dl considered diabetic [19, 20]. We recognize that a one-time capillary blood glucose measurement is not recommended for clinical use, though it has been shown to have sufficient sensitivity and specificity to diagnose diabetes in epidemiological population-based research [21] and is the recommended method for monitoring diabetes prevalence in the WHO’s STEP-wise approach to NCD risk factor surveillance [22].

Blood pressure was measured using established protocols in epidemiological studies [21, 22] and followed the WHO STEPWISE epidemiological hypertension protocol [23]. The participant did not have tea or coffee in the previous 10 min and was seated for at least 5 min prior to the test. Three readings (twice sitting and once standing) were taken in either arm at 5 min intervals with an OMRON digital blood pressure measuring device. The participants were considered as hypertensive if the mean of the three measurements were > 140 mmHg Systolic and/or > 90 mmHg Diastolic or where the participant reported having been diagnosed as hypertensive and receiving blood pressure-lowering treatment [24, 25]. It was not possible to return the next day for a further blood pressure measurement.

Depression and anxiety were assessed using the self-report Depression, Anxiety and Stress Scale (DASS) [26]. DASS is a set of three scales which separately measure depression, anxiety and stress. Participants were asked to use 4-point severity/frequency scales to rate the extent to which they experienced each state over the past week. Scores for depression, anxiety, and stress were calculated by summing the scores for the relevant items. Reliability of the three scales is considered adequate and test-retest reliability is likewise considered good with 0.71 for depression, 0.79 for anxiety, and 0.81 for stress [27]. The DASS anxiety scale correlates 0.81 with the Beck Anxiety Inventory, and the DASS Depression scale correlates 0.74 with the Beck Depression Scale [28]. DASS has previously been used as a valid and reliable measure of depression, anxiety and stress in India [29].

Socio–demographic variables included age, gender, income, literacy, occupation, education, marital status and religion. Behavioural risk factors included tobacco use, alcohol consumption, nutrition and physical activity. These risk factors were measured by standardized questions which have previously been validated in a prospective population cohort study in India [30] and can be found in Supplementary File 1. Additionally, health service utilisation was measured.

Social cohesion was measured using nine items from the Social Capital Community Benchmark Survey [31]. The items measured frequency of engagement in community life such as attendance at community meetings, religious events or clubs. Responses used a 5-point Likert scale and were summed to create a single scale (α = 0.87). As this tool had not previously been used in India, the items were adapted for cultural relevance by the authors, who established their content validity for use in Kerala. Wenger’s Practitioner Assessment of Network Type (PANT) was used to measure social network type [32]. PANT has previously been validated in a community population in India [33].

Physical and social functioning was measured by the 12-item interviewer-administered World Health Organisation Disability Assessment Schedule (WHODAS) 2.0 [34]. The WHODAS 2.0 covers six domains: understanding and communicating with the world; moving and getting around; self-care; getting along with people; life activities; and participation in society. Scores for each question range from zero (no difficulty) to four (extreme difficulty/cannot do). The standardized global score ranges from zero (non-disabled) to 100 (maximum disability). This measurement has been extensively validated in India and other low and middle-income countries [35].

All tools were available to the researchers without a requirement to purchase licenses. They were translated to local language (Malayalam), twice, by two researchers, and re-translated back into English before data entry. Items were queried to predict any possible issues in language or meaning within the particular ethnic community of each study site.

### Statistical analysis

All statistical analysis was performed using Mplus version 8 and SPSS version 24. Chi-squared tests were used to explore the characteristics of the sample by hypertensive and diabetic status, and gender. We performed latent class analysis incrementally to extract homogeneous latent classes of individuals based on their responses to social and behavioural risk factors in the survey. Based on the distribution of individual-level latent classes within catchment area population, distinct latent classes were identified to classify individuals at risk for chronic diseases management. Since most of our latent class indicators were order-categorical items, we employed nonparametric estimation not assuming normality. We used full-information maximum likelihood estimation, which allows for dependent variable missing data under missing at random assumptions, with the robust maximum likelihood estimator, which used model-based methods to accommodate our survey data.

To identify the best-fit model, we used the recommended four stage sequential modeling strategy [36]. In the first stage of the analyses, we estimated a series of traditional latent class models to determine the number of latent classes at the individual-level. Model fit of the competing models was compared using the Bayesian Information Criterion (BIC), where lower values indicate better model fit to the data. Classification quality of the competing models was assessed using entropy, a measure that summarizes how well the latent classes can be distinguished. Entropy values range from 0 to 1, with higher values indicating clearer distinctions among the latent classes. In addition, the mean class assignment probabilities equal to or larger than 0.8 was considered as a good class solution. At each stage, parsimonious solution (one of more very small classes) was considered in selecting a model with fewer classes. Additionally, models were evaluated and compared according to interpretability of the obtained solutions. A theoretically meaningful solution was preferred to uninterpretable solutions. All individuals with missing values were excluded in the regression analysis.

The socio-demographic characteristics of the three risk groups which emerged from the latent class analysis were compared using chi-squared tests. The relative contributions of the three risks groups to the diagnoses of diabetes and hypertension were explored using multinomial logistic regression analysis.

Finally, using structural equation models, we assessed the mediation effect of mental health indicators (depression or anxiety) and social or behavioural risk factors on high blood pressure and blood glucose. In the mediation analysis, the direct and indirect relationships was assessed, after controlling for all known confounders. A bias-corrected bootstrap method was used for drawing inference in mediation and moderation analysis.

## Results

### Characteristics of study population

A response rate of 97.8% was achieved in the survey. Table 1 reports descriptive information on the study population. The mean age of the participants was 53.6 years and almost two-thirds of the participants were women. Among 997 participants, 27% of the participants had no formal education or did not complete primary education. Approximately 58% of the participants were not engaged in any formal employment. The majority of the respondents were married and living with spouse. The prevalence of high blood pressure and blood glucose was 33 and 26% respectively. The prevalence of probable cases of depression and anxiety was 16 and 21% respectively. There were notable differences between men and women in the sample. Women were younger; had lower literacy levels; more likely to be working in the home; more likely to be widowed, divorced or separated; more likely to be living in smaller, less locally integrated networks; have a higher prevalence of depression and anxiety; and poorer physical and social functioning than men. However, rates of hypertension were higher in men than women (Table 1).

**Table 1:** Sample characteristics by hypertension, diabetes and gender of community-dwelling participants aged 30–90 in Keezhumadu, Kerala, India, 2018

	Not hypertensive	Hypertensive	^χ²^ ,*p* value	Not diabetic	Diabetic	^**χ2**^, ***p*** value	Male	Female	^**χ2**^, ***p*** value	Total
**Age**										
30–39	185 (22.8%)	18 (9.7%)	^χ2^ = 22.40, *p* < 0.001	188 (22.51%)	15 (9.3%)	^χ2^ = 35.33, *p* < 0.001	61 (16.7%)	142 (22.5%)	^χ2^ = 10.78, *p* = 0.029	203 (20.4%)
40–49	163 (20.1%)	39 (21.1%)		182 (21.80%)	20 (12.4%)		66 (18.1%)	136 (21.5%)		202 (20.3%)
50–59	173 (21.3%)	50 (27.0%)		171 (20.48%)	52 (32.1%)		81 (22.2%)	142 (22.5%)		223 (22.4%)
60–69	171 (21.1%)	57 (30.8%)		174 (20.84%)	54 (33.3%)		96 (26.3%)	132 (20.9%)		228 (22.9%)
70+	120 (14.8%)	21 (11.4%)		120 (14.37%)	21 (13.0%)		61 (16.7%)	80 (12.7%)		141 (14.1%)
**Gender**										
Male	276 (34.0%)	89 (48.1%)	^χ2^ = 12.94, p < 0.001	298 (35.7%)	67 (41.4%)	ns	–	–	–	365 (36.6%)
Female	536 (66.0%)	96 (51.9%)		537 (64.3%)	95 (58.6%)		–	–		632 (63.4%)
**Education**										
None	32 (3.9%)	9 (4.9%)	^χ2^ = 15.13, *p* = 0.004	32 (3.8%)	9 (5.6%)	^χ2^ = 20.80, *p* < 0.001	6 (1.6%)	35 (5.5%)	^χ2^ = 12.12, *p* = 0.016	41 (4.1%)
Did not complete primary	178 (21.9%)	49 (26.5%)		186 (22.3%)	41 (25.3%)		79 (21.6%)	148 (23.4%)		227 (22.8%)
Completed primary	243 (29.9%)	68 (36.8%)		246 (29.5%)	65 (40.1%)		126 (34.5%)	185 (29.3%)		311 (31.2%)
Completed secondary	176 (21.7%)	40 (21.6%)		182 (21.8%)	34 (21.0%)		85 (23.3%)	131 (20.7%)		216 (21.7%)
Completed tertiary	183 (22.5%)	19 (10.3%)		189 (22.6%)	13 (8.0%)		69 (18.9%)	133 (21.0%)		202 (20.3%)
**Literacy**										
Able to read newspaper	748 (92.1%)	174 (94.1%)	ns	774 (92.7%)	148 (91.4%)	ns	348 (95.3%)	574 (90.8%)	^χ2^ = 6.79, *p* = 0.009	922 (92.5%)
**Occupation**										
Paid work	266 (32.8%)	75 (40.5%)	ns	284 (34.0%)	57 (35.2%)	ns	224 (61.4%)	117 (18.5%)	^χ2^ = 305.2, *p* < 0.001	341 (34.2%)
Unemployed	206 (25.4%)	33 (17.8%)		195 (23.4%)	44 (27.2%)		69 (18.9%)	170 (26.9%)		239 (24.0%)
Housewife / husband	275 (33.9%)	62 (33.5%)		291 (34.9%)	46 (28.4%)		18 (4.9%)	319 (50.5%)		337 (33.8%)
Retired	65 (8.0%)	15 (8.1%)		65 (7.8%)	15 (9.3%)		54 (14.8%)	26 (4.1%)		80 (8.0%)
**Marital status**										
Never married	12 (1.5%)	6 (3.2%)	ns	16 (1.9%)	2 (1.2%)	ns	11 (3.0%)	7 (1.1%)	^χ2^ = 63.14, *p* < 0.001	18 (1.8%)
Married	675 (83.1%)	149 (80.5%)		696 (83.4%)	128 (79.0%)		340 (93.2%)	484 (76.6%)		824 (82.7%)
Widowed / divorced / separated	125 (15.4%)	30 (16.2%)		123 (14.7%)	32 (19.8%)		14 (3.8%)	141 (22.3%)		155 (15.6%)
**Social network typology**										
Family dependent	80 (9.9%)	18 (9.7%)	ns	86 (10.3%)	12 (7.4%)	ns	36 (9.9%)	62 (9.8%)	^χ2^ = 20.75, *p* < 0.001	98 (9.8%)
Locally integrated	215 (26.5%)	52 (28.1%)		220 (26.4%)	47 (29.0%)		125 (34.3%)	142 (22.5%)		267 (26.8%)
locally self-contained	79 (9.7%)	18 (9.7%)		76 (9.1%)	21 (13.0%)		28 (7.7%)	69 (10.9%)		97 (9.7%)
Wider community focus	208 (25.6%)	48 (26.0%)		210 (25.2%)	46 (28.4%)		94 (25.8%)	162 (25.6%)		256 (25.7%)
Private	230 (28.3%)	49 (26.5%)		243 (29.1%)	36 (22.2%)		82 (22.5%)	197 (31.2%)		279 (28.0%)
**Depression**										
non-case	683 (84.1%)	158 (85.4%)	ns	705 (84.4%)	136 (84.0%)	ns	334 (91.5%)	507 (80.2%)	^χ2^ = 22.33, *p* < 0.001	841 (84.4%)
probable case	129 (15.9%)	27 (15.0%)		130 (15.6%)	26 (16.1%)		31 (8.5%)	125 (19.8%)		156 (15.7%)
**Anxiety**										
non-case	636 (78.3%)	147 (79.5%)	ns	653 (78.2%)	130 (80.3%)	ns	321 (88.0%)	462 (73.1%)	^χ2^ = 30.24, *p* < 0.001	783 (78.5%)
probable case	176 (21.7%)	38 (20.5%)		182 (21.8%)	32 (19.8%)		44 (12.1%)	170 (26.9%)		214 (21.5%)
**Risk factor- smoking**										
Yes	102 (12.6%)	46 (24.9%)	^χ2^ = 18.04, *p* < 0.001	721 (86.4%)	128 (79.0%)	^χ2^ = 5.77, *p* = 0.016	132 (36.2%)	16 (2.5%)	^χ2^ = 207.04, *p* < 0.001	148 (14.8%)
No	710 (87.4%)	139 (75.1%)		114 (13.7%)	34 (21.0%)		233 (63.8%)	616 (97.5%)		849 (85.2%)
**Risk factor- Alcoholism**										
Yes	91 (11.2%)	39 (21.1%)	^χ2^ = 12.95, *p* < 0.001	105 (12.6%)	25 (15.4%)	ns	115 (31.5%)	15 (2.4%)	^χ2^ = 173.19, *p* < 0.001	130 (13.0%)
No	721 (88.8%)	146 (78.9%)		730 (87.4%)	137 (84.6%)		250 (68.5%)	617 (97.6%)		867 (86.9%)
**Told High Blood Pressure**										
No	568 (70.0%)	96 (51.9%)	^χ2^ = 22.08, *p* < 0.001	573 (68.6%)	91 (56.2%)	^χ2^ = 9.45, *p* = 0.002	270 (74.0%)	394 (62.3%)	^χ2^ = 14.07, *p* < 0.001	664 (66.6%)
Yes	244 (30.1%)	89 (48.1%)		262 (31.4%)	71 (43.8%)		95 (26.0%)	238 (37.7%)		333 (33.4%)
**Told Diabetes**										
No	616 (75.9%)	122 (66.0%)	^χ2^ = 7.70, *p* = 0.006	696 (83.4%)	42 (25.9%)	^χ2^ = 232.69, *p* < 0.001	272 (74.5%)	466 (73.7%)	^χ2^ = 14.07, *p* < 0.001	738 (74.0%)
Yes	196 (24.1%)	63 (34.1%)		139 (16.7%)	120 (74.1%)		93 (25.5%)	166 (26.3%)		259 (26.0%)
**WHODAS**										
Quartile 1	262 (32.3%)	57 (30.8%)	ns	278 (33.3%)	41 (25.3%)	ns	159 (43.6%)	160 (25.3%)	^χ2^ = 45.04, *p* < 0.001	319 (32.0%)
Quartile 2	153 (18.8%)	34 (18.4%)		160 (19.2%)	27 (16.7%)		70 (19.2%)	117 (18.5%)		187 (18.8%)
Quartile 3	206 (25.4%)	51 (27.6%)		211 (25.3%)	46 (28.4%)		82 (22.5%)	175 (27.7%)		257 (25.8%)
Quartile 4	191 (23.5%)	43 (23.2%)		186 (22.3%)	48 (29.6%)		54 (14.8%)	180 (28.5%)		234 (23.5%)

*Hypertensive* measured systolic blood pressure of > 140 mmHg or a measured diastolic blood pressure of > 90 mmHg

*Diabetic* blood glucose level > 200 mg/dl

*ns* not significant

Analysis undertaken = chi-squared tests

### Prevalence of social and lifestyle risk factors among participants with high blood pressure or blood glucose

The prevalence of one or more lifestyle (or behavioural) risk factor which could adversely affect NCD management was 38%. Of the participants who had an abnormally raised serum glucose, 30% were physically inactive, 15% were current smokers and 5% consumed alcohol regularly. Among participants with high blood pressure, 20% were physically inactive, 21% were current smokers and 7% consumed alcohol regularly.

The prevalence of one or more social risk factors was 70%. Among participants with high blood glucose, 10% reported that they could neither read nor write, 43% had a low income, 11% had high stress, 31% resided in a community with poor social cohesion and 22% had a poor social support network. Among participants with raised blood pressure, 11% reported that they could not read or write, 48% had poor income, 13% had high stress, 27% resided in community with poor social cohesion and 28% had disintegrated social support network.

### Effect of social, behavioural and mental health conditions on blood pressure and blood glucose

In the latent class analysis, social and behavioural risk factors were pooled together to identify homogeneous sub-groups of participants with differential risks for chronic diseases management. The best fit model was identified using the Akaike Information Criterion (AIC), BIC and entropy measure. According to the model fit parameters and theory, BIC gradually reduced from the baseline model to the three-class model, and then began to rise with the four-class model. Hence the three-class model was selected as the best fitting model with the smallest BIC value and high entropy (Table S1, supplementary file 2).

On the basis of the estimated conditional response probabilities, participants assigned to Class 1 (20%) had lower probability for physical inactivity, higher probability for being a current smoker or regular consumer of alcohol and meat, and lower probability for stress. Given the predominance of behavioural characteristics in class 1, this is considered as a ‘behaviourally’ at-risk group.

Class 2 (15%) was similar to class 1, but there were distinct social and other risk factors. It was characterised by higher probability for physical inactivity, higher probability for illiteracy, stress, low income and disintegrated social support network types. Given the predominance of social risk characteristics, this group is considered as a ‘socially’ at-risk group.

**Fig. 1:**
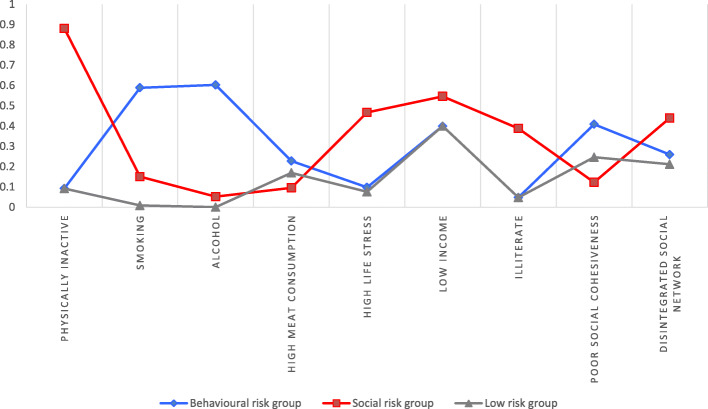
Homogeneous sub-groups with social and behavioural risk factors for diabetes and hypertension in community-dwelling participants aged 30–90 in Keezhumadu, Kerala, India, 2018. Class 1 = behavioural risk group. Class 2 = social risk group. Class 3 = low risk group. We performed a latent class analysis which used full-information maximum likelihood estimation, allowing for dependent variable missing data under missing at random assumptions, with the robust maximum likelihood estimator which used model-based methods to accommodate our survey data

Class 3 (64%) was a low risk group as it was characterised by a lower probability for physical inactivity, illiteracy, low income, behavioural risks (smoking and alcohol) and stressful life (Fig. 1).

In comparison with the low risk group, the distribution of mean scores for physical and mental health differed in the behavioural (class 1) and social risk (class 2) groups. The mean systolic blood pressure in the behavioural risk group was 137.9, social risk group was 134.0 and low risk group was 130.4. The mean blood glucose in the behavioural risk group was 161.9, social risk group was 157.2, and low risk group was 150.1. The mean score (s.d) for probable common mental health conditions (depression and anxiety) in the behavioural risk group was 4.7 (9.3), social risk group was 14.2 (9.3) and low risk group was 5.5 (4.9). However, after controlling for age and sex, the difference was no longer significant for serum glucose.

The socio-demographic features of the behavioural and social risk groups were also distinct (Table 2). The behavioural risk group was characterized by male sex and married status. They were mostly employed and had above median income. In contrast, the social risk group were predominantly women, over the age of 60, a high proportion of whom were not married, and most who had below median income and were either not employed or a househusband/wife. Most had low levels of education but, by contrast, high levels of disability.

**Table 2:** Socio-demographic characteristics of risk groups in community-dwelling participants aged 30–90 in Keezhumadu, Kerala, India, 2018

	CLASS 1 Behavioural risk group	CLASS 2 Social risk group	CLASS 3 Low risk group	^**χ2**^, ***p*** value
**Age**				
30–39	34 (19.5%)	9 (6.5%)	160 (23.4%)	^χ2^ = 86.91, *p* < 0.001
40–49	28 (16.1%)	18 (13.0%)	156 (22.8%)	
50–59	43 (24.7%)	22 (15.8%)	158 (23.1%)	
60–69	47 (27.0%)	40 (28.8%)	141 (20.6%)	
70+	22 (12.6%)	50 (36.0%)	69 (10.1%)	
**Gender**				
Male	157 (90.2%)	31 (22.3%)	177 (25.9%)	^χ2^ = 261.78, *p* < 0.001
Female	17 (9.8%)	108 (77.7%)	507 (74.1%)	
**Marital status**				
Married	162 (93.1%)	79 (56.8%)	583 (85.2%)	^χ2^ = 81.04, *p* < 0.001
Unmarried / widowed / separated	12 (6.9%)	60 (43.2%)	101 (14.8%)	
**Income**				
Above median	109 (62.6%)	67 (48.2%)	404 (59.1%)	^χ2^ = 7.33, *p* = 0.026
Below median	65 (37.4%)	72 (51.8%)	280 (40.9%)	
**Wenger social network**				
Integrated social network	4 (2.3%)	5 (3.6%)	27 (4.0%)	ns
Non-integrated social network	170 (97.7%)	134 (96.4%)	657 (96.1%)	
**Occupation**				
Unemployed	25 (14.4%)	54 (38.9%)	160 (23.4%)	^χ2^ = 163.59, *p* < 0.001
Employed	118 (67.8%)	22 (15.8%)	201 (29.4%)	
Housewife / husband	6 (3.5%)	54 (38.9%)	277 (40.5%)	
Retired	25 (14.4%)	9 (6.5%)	46 (6.7%)	
**Education**				
Completed primary	36 (20.7%)	88 (63.3%)	144 (21.1%)	^χ2^ = 119.89, *p* < 0.001
Completed secondary	111 (63.8%)	44 (31.7%)	372 (54.4%)	
Completed tertiary	27 (15.5%)	7 (5.0%)	168 (24.6%)	
**Disability**				
1st quartile	83 (47.7%)	1 (0.7%)	235 (34.4%)	^χ2^ = 334.26, *p* < 0.001
2nd quartile	36 (20.7%)	4 (2.9%)	147 (21.5%)	
3rd quartile	38 (21.8%)	19 (13.7%)	200 (29.2%)	
4^th^ quartile	17 (9.8%)	115 (82.7%)	102 (14.9%)	
**Hypertension (diagnosed)**				
No	119 (68.4%)	117 (84.2%)	576 (84.2%)	^χ2^ = 23.77, *p* < 0.001
Yes	55 (31.6%)	22 (15.8%)	108 (15.8%)	
**Diabetes (diagnosed)**				
No	140 (80.4%)	112 (80.5%)	586 (85.7%)	ns
Yes	34 (19.5%)	27 (19.4%)	98 (14.3%)	

Social, behavioural and low risk was categorized based on the latent class analysis of the different risk variables. The variables used for the analysis are physical activity, diet, smoking, alcoholism, stress, social cohesion, social network, income and literacy

*Hypertensive* measured systolic blood pressure of > 140 mmHg or a measured diastolic blood pressure of > 90 mmHg

*Diabetic* blood glucose level > 200 mg/dl

Analysis undertaken = chi-squared tests

*ns* not significant

The behavioural and social risk groups did not independently increase the risk of diabetes, though the behavioural risk group had an independent relationship with an increased risk of hypertension (Table 3). Social and behavioural risks are highly gendered (Table 2) and are likely to be influenced by other variables, such as mental health problems, to increase the risk of diabetes and hypertension.

**Table 3:** Multinomial logistic regression analysis of behavioural and social risk groups with diagnosed diabetes and hypertension in community-dwelling participants aged 30–90 in Keezhumadu, Kerala, India, 2018

	Diagnosed Diabetic	Diagnosed Hypertensive
	Relative risk ratio (95% CI), *p* value	Relative risk ratio (95% CI), *p* value
Class Groups		
Low risk group (comparator)	1	1
Behavioural risk group	1.45 (0.94–2.24), *p* = 0.090	2.46 (1.69–3.60), *p* < 0.001
Social risk group	1.44 (0.90–2.31), *p* = 0.128	1.00 (0.61–1.65), *p* = 0.991

Relative risk ratios are presented with 95% CI as coefficients of the regression model. The low risk group is the comparison variable

*Hypertensive* measured systolic blood pressure of > 140 mmHg or a measured diastolic blood pressure of > 90 mmHg

*Diabetic* blood glucose level > 200 mg/dl

**Fig. 2:**
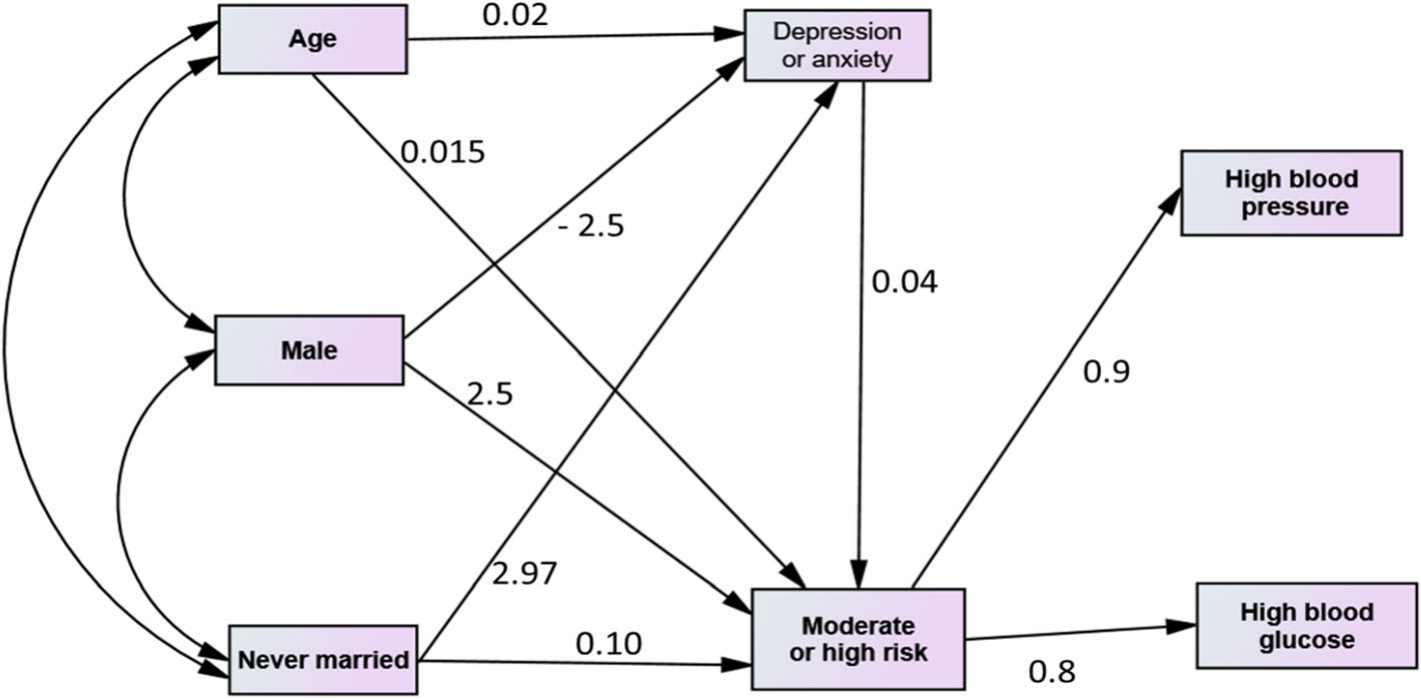
Structural equation modelling showing the direct and indirect effect of common mental health conditions on high blood pressure and blood glucose in community-dwelling participants aged 30–90 in Keezhumadu, Kerala, India, 2018. Structural equation modelling analysed the mediation effect (direct and indirect) of depression and anxiety, and the behavioural and social risk groups, on hypertension and diabetes. A bias-corrected bootstrap method was used for drawing inference in mediation and moderation analysis. The regression coefficients are shown in the figure, indicating the strength and direction of effect

The results of serial multiple mediators modelling of the relationships between the common mental health conditions and social and behavioural risk factors are shown in Fig. 2. This allowed simultaneous testing of each mediation mechanism by which demographic, social and behavioural risk factors and mental health conditions influence high blood pressure and high blood glucose, whilst accounting for the shared association between mediators and other personal characteristics. After controlling for all known covariates, the effects of age, female sex, and marital status on high blood pressure and high glucose were mediated by social and behavioural risk factors. In the analysis, depression and anxiety was not directly associated with increased blood glucose or blood pressure. Mental health indicators operated through both social and behavioural risk factors.

## Discussion

The high rates of hypertension found in this study were consistent with the documented high rates found in urban areas, higher socio-economic status groups and in southern states in India [7, 37, 38]. The raised blood glucose levels were significantly higher than is previously reported in the literature [39] and hypertension rates were also slightly higher than in other studies [40], highlighting the high prevalence of risk factors for NCDs in Kerala. The prevalence of depression and anxiety in our sample was comparable to other studies in south India [41, 42].

This study has demonstrated significant relationships between the presence of social and behavioural risk factors and the poor management of elevated serum glucose and high blood pressure in this South Indian community. This relationship has not been reported in the Indian context. There is a recommendation-implementation gap in NCD risk factor management in India and several putative reasons have been considered. Until now, however, data related to social factors which negatively affect the management of NCDs has not been studied in detail. This study adds to the findings of other, earlier studies (e.g. [43–45]).

Our analysis has demonstrated two distinct clusters in people with hypertension and/or glucose intolerance. These can be classified as follows: class 1, the behavioural risk factor group with adverse lifestyle activities such as smoking and excess alcohol ingestion, tends to be economically stable and has stronger family and community connections. Class 2, the social risk factor group, constituted 15% of this sample. Those in this group exhibit physical inactivity, high stress levels, low income, poor literacy, low social cohesion, a disintegrated social support network and the highest prevalence of depression and anxiety in the sample as a whole. The clustering of factors described above which appears to adversely affect NCD management has, to our knowledge, not been found before in India.

It has been observed in this study that the effect of depression and anxiety symptoms on elevated serum glucose and hypertension control is related to social and behavioural risk factors, predominantly in older, never-married females (Fig. 2). Culturally, these women tend to be house-bound and consequently have low levels of exercise. Also, depression and anxiety could limit the relationships these women have with other members of the local community, as mental health issues carry a stigma in India [46], thereby reducing their opportunity for physical exercise. Independent of these factors, depression and anxiety reduces interest in undertaking physical exercise [47]. This indicates that future interventions should address both mental health needs and the social context of those who have glucose intolerance or are hypertensive.

The social risk group has the lowest income and education levels in the sample. Poverty and social exclusion of this group is compounded by poor connections with the local community. Neighbourhood connections have been found to play an important role in NCD risk factor management and perceived health of Indians [8, 9]. Neighbourhoods and social connections are linked to many issues such as socio-economic status, culture, social exclusion and physical environment, which impact on health directly (access to services) as well as indirectly (prevention – access to facilities for exercise or transport). The social risk group could represent a group left behind by economic growth in India, which suggests that the increase in cardiovascular risk factors in Kerala may be related to growing social inequality, affecting both those in poverty and those with increasing wealth.

### Limitations of the study

It is possible that there are social and other risk factors that impact on risk factor management and which we have not measured. However, we have endeavoured to measure the key known social risk factors in relation to NCDs as informed by our scoping review [15]. In addition, we have used measures which have been widely validated in India.

Our data may be indicative of factors not measured in the survey, but we aim to remedy this in a future in–depth qualitative study within the same catchment area which will include participants from each risk group.

Due to the fact that the data was collected in a semi-rural geographical area we acknowledge that further research in either urban or purely rural areas is required. Kerala has a higher life expectancy and better education and health systems than many states in India which reduces its generalizability. However, while our sample may not be indicative of the rest of India, the higher prevalence of NCDs in south India is nevertheless of scholarly and practice importance.

### Implications for practice and research

These findings should be addressed in larger studies, possibly linked to complex interventions, which will elucidate many of the issues that remain unknown. What this study does highlight is that the social factors, which are increasingly being recognized as key to improved NCD management, are complex and the interactions of these with traditional risk factors for NCD need to be further investigated.

In India community health workers (Accredited Social Health Workers or ASHAs) play an essential community role. While used initially for maternal and child health, their role in chronic disease management has been tested. Since 2010, the National Program for the Prevention and Control of Cardiovascular Diseases, Diabetes, Cancer and Stroke has tested the use of ASHAs in 100 pilot districts in 21 States of India in the prevention and management of chronic conditions such as diabetes and hypertension, and their ability to be trained to address hypertension and diabetes has been shown to be positive [48]. ASHAs, however, have no training in evaluation of the social risk factors for the poor management of chronic disease, but have the trust of the community and an integral knowledge of families and persons under their care. They are considered as a ‘gateway’ to the communities in which they work, as they often come from these communities.

While interventions to manage behavioural risk are located within the health system, social issues are usually managed within social care or community services. The interaction of these two service delivery systems, i.e. medical and social, represents an opportunity to integrate two powerful but different approaches to prevention and management of risk factors for NCD. Medicine approaches health issues in a linear paradigm using a diagnostic, intervention and outcome approach while social science operates from a systemic networked framework of understanding. These approaches need to be integrated if prevention and intervention is to be effective [49]. For example, case management skills may be required to prioritise those in greatest social need who may require additional support to access and engage with local health services or make changes to their lifestyle. Interventions need to include strategies to manage mental health issues in addition to making lifestyle changes.

The integration of medical and social interventions within the public health system in Kerala could be complex as primary health care currently has gaps in infrastructure and staffing, and variable service quality. Lack of critical drugs, outdated diagnostic tools, paucity of trained medical personnel and inaccessible healthcare facility are also major barriers for health care utilization. The burden is heavy and the simultaneous presence of communicable diseases and lifestyle diseases together with marginalization of poor people, uncontrolled growth of the private sector and escalation of health care costs, makes health, for many people in India, an unaffordable commodity [50].

## Conclusions

From our data there are two distinct clusters of behavioural and social factors associated with the suboptimal management of hypertension and diabetes in the Indian context. Depression and anxiety were also found to be associated with abnormally increased blood glucose or blood pressure, though mediated through the social and behavioural risk factors. The clustering of risk factors found in this survey raise significant questions that related to optimal management of NCD risk. Other research is needed to replicate this study in Kerala and elsewhere in India. If our findings are replicated it will suggest a different approach to the management of risk factors for CVD which incorporates social work into primary health care. Targeting social risk factors can reduce behavioural risk factors, while interventions which solely address behavioural risk factors are less likely to manage social risk factors.

## Supplementary information

**Additional file 1.** Behaviour and lifestyles questionnaire. Questionnaire used to collect data on behaviour and lifestyle.

**Additional file 2.** Table S1 Four stage sequential modeling strategy to compare the Information fit criteria of the latent class models. Table highlighting method of selection of number of latent classes.

## Data Availability

The dataset used in the current study is available from the corresponding author on reasonable request.

## Abbreviations

AIC: Akaike information criterion
ASHA: Accredited social health worker
BIC: Bayesian information criterion
CVD: Cardiovascular disease
DASS: Depression, anxiety and stress scale
LMIC: Low and middle-income country
NCD: Non-communicable disease
PANT: Practitioner assessment of network type
UKIERI: UK-India education and research initiative
WHODAS: World Health Organisation disability assessment schedule
