# Supplementary material for: Social and behavioural risk factors in the prevention and management of cardiovascular disease in Kerala, India: a catchment area population survey
Source: BMC Cardiovasc Disord. 2020 Jul 8;20:327. doi: 10.1186/s12872-020-01595-x (PMC7346640; doi:10.1186/s12872-020-01595-x)
Supplement: Supplementary file 1 — Additional file 1. Behaviour and lifestyles questionnaire. Questionnaire used to collect data on behaviour and lifestyle. [file 12872_2020_1595_MOESM1_ESM.docx]

**Supplementary File 1**

**BEHAVIOUR AND LIFESTYLES QUESTIONNAIRE**

**1.1 TOBACCO USE**

| 1.1.1 | Has there ever been a period when you smoked cigarettes, cigars, or a pipe, chewing tobacco or snuff nearly every day? | No 0  Yes 1 |
| --- | --- | --- |
| **If no, skip to Q 9.2** | | |
| 1.1.2 | How old were you when you started using tobacco regularly? |  |
| 1.1.3 | Do you still use tobacco daily? | No 0  Yes 1 |
| **If yes, skip to 9.1.5** | | |
| 1.1.4 | How old were you when you stopped? |  |
| 1.1.5 | What did you smoke? | Manufactured cigarettes 1  Hand-rolled cigarettes 2  Pipes full of tobacco 3  Cigars 4  Others 5 |
| 1.1.6 | On average, how many times a day do you use it |  |
| 1.1.7 | Do you currently use smokeless tobacco products  Daily (snuff)? | No 0  Yes 1 |
| 1.1.9 | In the past, did you ever use smokeless tobacco such  as [snuff, chewing tobacco, or betel] daily? | No 0  Yes 1 |
| 1.1.10 | What did you use? | Snuff, by mouth  Snuff, by nose  Chewing tobacco  Betel quid  others |
| 1.1.11 | On average, how many times a day do you use it |  |
| 1.1.6 | Did someone in your home smoke when you were  present? | No 0  Yes 1 |
| 1.1.7 | Did someone smoke in closed areas in your workplace (in the building, in a work area or a specific office) when you were present? | No 1  Yes 1 |

**1.2. ALCOHOL**

| 1.2.1 | Have you ever consumed an alcoholic drink? | No 0  Yes 1 |
| --- | --- | --- |
| **If never a drinker, skip to 9.3** | | |
| 1.2.2 | During the past 12 months, how frequently have you  had at least one alcoholic drink? | Daily 1  5-6 days per week 2  1-4 days per week 3  1-3 days per month 4  Less than once a month 5 |
| 1.2.3 | What was the most you would drink in an average week?  (Record maximum regular consumption in UNITS of alcohol per week)  1 unit = a small glass of beer  a single measure of spirit 1  1 glass of wine or sherry  32 units= 1 bottle of spirit -2  Don’t know -3 |  |
| 1.2.4 | What about current drinking? |  |
| 1.2.5 | Has there ever been a period of several years when you would have said that you were a heavy drinker? | No 0  Yes 1 |
| 1.2.6 | Have you ever had treatment or help for drinking from a doctor or some other agency? | No 0  Yes 1 |

**1.3 DIET**

| 1.3.1 | How often do you eat meat? | Never 0  Some days 1  Most days 2  Every day 3 |
| --- | --- | --- |
| 1.3.2 | How often do you eat fish? | Never 0  Some days 1  Most days 2  Every day 3 |
| 1.3.3 | How many servings of fruit and vegetables have you eaten over the last 3 days? |  |
| 1.3.4 | Do you ever go hungry because there is not enough food to eat?  How often does this happen? | Never 0  Some days 1  Most days 2  Every day 3 |
| 1.3.5 | On average, how many meals per week do you eat that were not prepared at a home? |  |

**1.4 PHYSICAL ACTIVITY**

| 1.4.1 | Taking into account both work and leisure, would you say that you are | very physically active 1  fairly physically active 2  not very physically active 3  not at all physically active 4 |
| --- | --- | --- |
| 1.4.2 | Does your work involve vigorous-intensity activity that causes large increases in breathing or heart rate like [carrying or lifting heavy loads, digging or construction work] for at least 10 minutes continuously? | No 0  Yes 1 |
| 1.4.3 | How much time do you spend doing vigorous-intensity activities at work on a typical day? |  |
| 1.4.4 | Does your work involve moderate-intensity activity that causes small increases in breathing or heart rate such as brisk walking [or carrying light loads] for at least 10minutes continuously? | No 0  Yes 1 |
| 1.4.5 | How much time do you spend doing moderate-intensity activities at work on a typical day? |  |
| 1.4.6 | Do you walk or use a bicycle (pedal cycle) for at least  10 minutes continuously to get to and from places? |  |
| 1.4.7 | Do you do any vigorous-intensity sports, fitness or  recreational (leisure) activities that cause large  increases in breathing or heart rate like [running or  Football] for at least 10 minutes continuously? | No 0  Yes 1 |
| 1.4.8 | How much time do you spend doing vigorous-intensity sports, fitness or recreational activities on a typical day? |  |
| 1.4.9 | Do you do any moderate-intensity sports, fitness or  recreational (leisure) activities that cause a small  increase in breathing or heart rate such as brisk  Walking, [cycling, swimming, volleyball] for at least minutes continuously? | No 0  Yes 1 |
| 1.4.10 | How much time do you spend doing moderate-intensity sports, fitness or recreational (leisure) activities on a typical day? |  |
| 1.4.11 | How much time do you usually spend sitting or reclining on a typical day? |  |
| 1.4.12 | All in all, do you exercise…….. than you used to do ten years ago? | more 3  as much 2  or less 1 |
