# Supplementary material for: Social and behavioural risk factors in the prevention and management of cardiovascular disease in Kerala, India: a catchment area population survey
Source: BMC Cardiovasc Disord. 2020 Jul 8;20:327. doi: 10.1186/s12872-020-01595-x (PMC7346640; doi:10.1186/s12872-020-01595-x)
Supplement: Supplementary file 2 — Additional file 2. Table S1 Four stage sequential modeling strategy to compare the Information fit criteria of the latent class models. Table highlighting method of selection of number of latent classes. [file 12872_2020_1595_MOESM2_ESM.docx]

**Supplementary file 2**

**Table S1** **Four stage sequential modeling strategy to compare the information fit criteria of the latent class models**

| Latent class | AIC | BIC | Adjusted BIC | Entropy |
| --- | --- | --- | --- | --- |
| 2 latent classes | 10489.27 | 10602.08 | 10529.03 | 0.55 |
| 3 latent classes | 10292.31 | 10463.98 | 10352.82 | 0.80 |
| 4 latent classes | 10314.51 | 10496.03 | 10387.76 | 0.71 |

Sequential modelling was used to determine the number of latent classes at the individual-level. Model fit of the competing models was compared using the Bayesian Information Criterion (BIC), where lower values indicate better model fit to the data. Classification quality of the competing models was assessed using entropy, a measure that summarizes how well the latent classes can be distinguished. Entropy values range from 0 to 1, with higher values indicating clearer distinctions among the latent classes. In addition, the mean class assignment probabilities equal to or larger than 0.8 was considered as a good class solution. At each stage, parsimonious solution (one or more very small classes) was considered in selecting a model with fewer classes.

AIC: Akaike Information Criterion

BIC: Bayesian Information Criterion
